# *Haemophilus influenzae* Type b Meningitis in Infants, New York, New York, USA, 2022–2023

**DOI:** 10.3201/eid3103.240946

**Published:** 2025-03

**Authors:** Anne Ewing, Sydney Haldeman, Megan J. Job, Caitlin Otto, Adam J. Ratner

**Affiliations:** New York University Grossman School of Medicine, New York, New York, USA

**Keywords:** *Haemophilus influenzae* type b, meningitis/encephalitis, bacteria, infants, whole-genome sequencing, epidemiology, New York

## Abstract

Two unvaccinated infants residing in the same borough of New York, New York, USA, had *Haemophilus influenzae* type b meningitis develop 1 year apart. Whole-genome sequencing and phylogenetic analysis revealed the isolates shared a previously undescribed multilocus sequence type and were more closely related to each other than to other sequenced strains.

*Haemophilus influenzae* type b (Hib) invasive infections and upper respiratory tract colonization have declined since the introduction of polysaccharide conjugate Hib vaccines, but sporadic cases still occur, leading to serious illness or death, especially in young children (*1*,*2*). Undervaccinated communities can serve as reservoirs for Hib colonization. Prior investigations of invasive Hib case clusters identified the same multilocus sequence type (ST) in multiple cases within undervaccinated Amish communities 14 years apart (*3*). Our medical center in New York, New York, USA, received 2 unvaccinated infants with Hib meningitis in 2022–2023, both of whom resided in the same borough, prompting further investigation.

## The Study

Case-patient 1, a 3-month-old, previously healthy, unvaccinated girl, came to our facility with 2 days of fever and lethargy. Physical examination revealed a full fontanelle, right upward gaze deviation, and focal seizure activity. She required noninvasive respiratory support for hypoxia and fluid resuscitation for septic shock. We administered vancomycin and ceftriaxone and admitted her to the intensive care unit. Blood and cerebrospinal fluid (CSF) cultures grew *H. influenzae*, later identified by the New York City Department of Health and Mental Hygiene as type b. Complications impeding treatment included seizures and bilateral subdural empyemas, requiring surgical drainage. She completed 4 weeks of ceftriaxone with clinical improvement and returned home on a continuing course of antiepileptic medication.

Case-patient 2, a 5-month-old, unvaccinated boy, came to our facility with a history of prematurity and 3 days of fever, lethargy, and acute perioral cyanosis. He was in septic shock, requiring vasopressor support with hypoxic respiratory failure and encephalopathy requiring endotracheal intubation. Shortly after admission to the intensive care unit, the patient’s pupils became fixed and dilated. Computed tomographic imaging of the brain revealed diffuse cerebral edema. Blood and CSF cultures grew *H. influenzae*, later identified as Hib by New York City Department of Health and Mental Hygiene. A multiplex PCR panel from the nasopharynx detected rhinovirus and enterovirus. The patient underwent initial treatment with linezolid and cefepime and then transitioned to ceftriaxone for 10 days. He also completed a 7-day course of metronidazole for presumed aspiration pneumonia. His severe neurologic injury with absence of brain stem reflexes did not improve. After 1 month of hospitalization, the patient transitioned to a rehabilitation facility, still requiring invasive mechanical ventilation.

We performed a retrospective chart review of pediatric patients (0–5 years of age) with a sterile-site culture positive for Hib who were seen in our health system during January 1, 2013–December 31, 2023. We also conducted whole-genome sequencing of Hib isolates from the 2 case-patients in this study. We isolated bacterial DNA as previously described and performed short-read sequencing on the NovaSeq 6000 platform (Illumina, https://www.illumina.com) for both the blood and CSF isolates from the 2 patients (*4*). We performed long-read sequencing on the blood isolates by using the Oxford Nanopore Technologies platform R10.4.1 (Oxford Nanopore Technologies, https://nanoporetech.com), conducting hybrid assembly with Trycycler (https://github.com/rrwick/Trycycler) and polishing with polypolish 0.6.0 (https://github.com/rrwick/Polypolish), resulting in closed, error-corrected genomes (*5*,*6*). We submitted genome sequences to the National Center for Biotechnology Information (https://www.ncbi.nlm.nih.gov) (GenBank accession nos. CP148001 and CP148002). We determined multilocus sequence types (STs) by using the PubMLST server (*7*). We detected single-nucleotide polymorphisms and insertions/deletions with snippy 4.6.0 (https://software.cqls.oregonstate.edu/updates/snippy-4.6.0). We downloaded all available *H. influenzae* genome sequences from GenBank (n = 2,199) by using National Center for Biotechnology Information Datasets command line tools 16.0.0 (https://www.ncbi.nlm.nih.gov/datasets/docs/V2). In silico serotype prediction using hicap 1.0.3 (https://github.com/scwatts/hicap) generated a dataset of 73 nonredundant Hib genomes (*8*). We constructed a genetic distance tree, including those 73 genomes plus the 2 newly generated genomes by using mashtree 1.2.0 and visualized that tree in Microreact (*9*,*10*).

The 2 cases we describe were the only invasive pediatric (0–5 years of age) cases of Hib identified at our institution during the 10-year span we investigated. We obtained antibiotic susceptibility data for the 2 cases we studied (Appendix Table 1) and generated high-quality closed genome sequences from the 2 Hib isolates. Both strains belonged to a previously unreported ST, newly assigned the identifier ST2832 within the ST6 clonal complex (Appendix Table 2). Analysis with hicap confirmed that each isolate contained 2 copies of the type b capsule locus, one of which had a truncation in the *bexA* gene (Appendix Figure). In a comparative genomic analysis of the 2 ST2832 isolates and the 73 genomes in our dataset, we enumerated core single-nucleotide polymorphisms among the Hib strains (Appendix Table 2). We also performed short-read sequencing on matched CSF isolates for each case and detected no variants between the blood and CSF isolates within either individual case. A genetic distance tree (Figure) demonstrated that the 2 ST2832 isolates from this study were more closely related to each other than to any other Hib genomes in the dataset.

**Figure:**
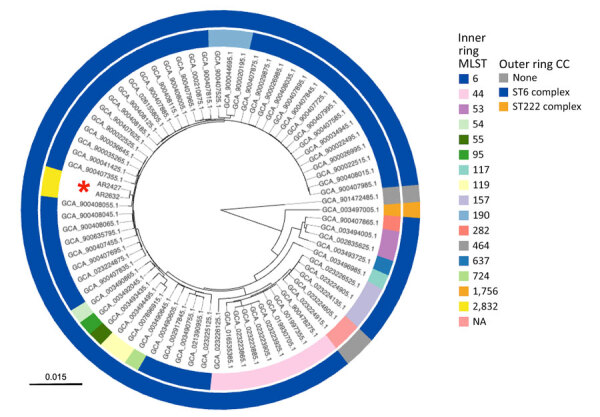
Genetic distance tree constructed to assess genetic relatedness among strains in study of *Haemophilus influenzae* type b (Hib) meningitis in infants, New York, New York, USA, 2022–2023. We constructed the tree using Mashtree *(**10**)* for Hib strains from the 2 New York patients (red asterisk) and reference sequences from GenBank (Appendix Table). Rings are color-coded to indicate MLST (inner ring) and CC (outer ring). Tree was rooted using the genome for NCTC 8468 (GenBank accession nos. GCA_90147285.1), a division II, *sodC*-containing Hib strain distantly related to other sequenced Hib isolates. Branch lengths represent mash distances. CC, clonal complex; MLST, multilocus sequence type; NA, not available.

Conjugate vaccination programs have been highly successful in decreasing the burden of invasive Hib disease, in part through reduction of nasopharyngeal carriage among vaccine recipients (*2*,*11*). However, even in the setting of widespread vaccination, sporadic cases occur among unvaccinated infants, older adults, and immunocompromised patients (*1*,*2*,*12*). Among children born in New York, New York, in 2021, less than 65% received their primary series of Hib vaccination on schedule (by 7 months of age), despite eventual coverage of ≈90% by 13 months of age (*13*). The basic reproductive rate of Hib has been estimated to be 3.3, implying a target immunization rate of ≥70% for disease control (*14*). Adequate primary series vaccination, boosters, and catchup programs are critical for herd immunity. Susceptible infants acquire Hib from colonized persons (generally young children), and such colonization is more common within underimmunized communities (*11*). Thus, sporadic invasive Hib cases, especially with related strains, may be sentinel events that indicate increased colonization within local populations, possibly owing to decreased immunization rates.

Limitations of our analysis include the small number of identified strains and limited whole-genome sequencing data from invasive Hib strains in the United States, requiring our comparisons to include both colonizing and invasive strains. Our observations are reminiscent of a report of 3 unvaccinated children hospitalized with invasive Hib disease within 5 months in 2014 (*3*). Two of those children were from the same Amish community and had Hib strains with a shared ST (ST45), described 14 years earlier in Amish communities in Pennsylvania, USA.

Vaccination rates are not the sole determinants of community levels of Hib carriage. In some populations with adequate vaccination coverage, factors like crowding might contribute to high colonization rates. Nolen et al. described 33 cases of invasive Hib over 14 years in Alaska, 27 of which were in Alaska native children, all originating from high-density indigenous communities despite ≈90% vaccination coverage in that population (*15*). That research group identified multiple distinct Hib STs in their analysis, implicating factors other than clonal spread in Hib carriage. Robust local surveillance of Hib vaccination coverage and routine whole-genome sequencing of isolates from invasive Hib disease would aid early identification of inadequate herd immunity and potential outbreaks. Studies of Hib colonization, particularly in communities with low uptake of routine childhood vaccinations, are urgently needed, as are ongoing efforts to combat vaccine hesitancy.

## Conclusions

We identified Hib meningitis in 2 geographically linked, unimmunized infants over the course of ≈1 year. Our findings, including the newly described ST and the relatedness of the 2 isolates, suggest ongoing colonization and transmission of this strain in New York communities. Despite the small number of cases described in this report, our findings raise concern for ongoing transmission of potentially virulent Hib strains in New York, New York, placing unvaccinated children at risk.

Appendix Additional information for *Haemophilus influenzae* type b meningitis in infants, New York, New York, USA, 2022–2023.
