# Supplementary material for: Haemophilus influenzae Type b Meningitis in Infants, New York, New York, USA, 2022–2023
Source: Emerg Infect Dis. 2025 Mar;31(3):579–82. doi: 10.3201/eid3103.240946 (PMC11878314; doi:10.3201/eid3103.240946)
Supplement: Appendix — Additional information for Haemophilus influenzae type b meningitis in infants, New York, New York, USA, 2022–2023. [file 24-0946-Techapp-s1.pdf]

Article DOI: http://doi.org/10.3201/eid3103.240946

EID cannot ensure accessibility for supplementary materials supplied by authors. Readers who have difficulty accessing supplementary content should contact the authors for assistance.

Haemophilus influenzae Type b Meningitis in Infants, New York, New York, USA, 2022–2023

Appendix

Appendix Table 1. Antimicrobial susceptibility data obtained by using Kirby-Bauer disk diffusion for Hib isolates from 2 cases in a study of *Haemophilus influenzae* type b meningitis in infants, New York, NY, USA, 2022–2023*

Case	Test Source	Ampicillin	Ceftriaxone	Levofloxacin	TMP-SMX
**Case 1**	**Blood**	Susceptible	Susceptible	Susceptible	Resistant
	**CSF**	Susceptible	Susceptible	ND	Intermediate
**Case 2**	**Blood**	Intermediate	Susceptible	Susceptible	Resistant
	**CSF**	Susceptible	Susceptible	Susceptible	Resistant

* CSF: cerebrospinal fluid; Hib: *Haemophilus influenzae* type b: ND: not determined; TMP-SMX: trimethoprim-sulfamethoxazole.

Appendix Table 2. Hib genomes included in comparative genomic analysis and corresponding multilocus sequence type, clonal complex, allelic profiles, and selected metadata from NCBI/GenBank for a study of *Haemophilus influenzae* type b meningitis in infants, New York, NY, USA, 2022–2023*

Strain ID	MLST	CC	MLST alleles							Collection Date	Source	Location	Core SNPs
			adk	*atpG*	*frdB*	*fucK*	*mdh*	*pgi*	*recA*				
AR2427 (CP148002)	2832	ST-6 complex	10	14	15	5	4	7	8	2022	blood	USA: New York, NY	0
AR2632 (CP148001)	2832	ST-6 complex	10	14	15	5	4	7	8	2023	blood	USA: New York, NY	197
GCA_000210875.1	6	ST-6 complex	10	14	4	5	4	7	8	NA	NA	NA	2589
GCA_001997355.1	44	ST-6 complex	10	14	4	3	4	3	8	NA	NA	NA	10594
GCA_002835625.1	53	ST-6 complex	10	14	5	7	4	7	8	2017	NA	India	15761
GCA_002917845.1	6	ST-6 complex	10	14	4	5	4	7	8	2017	blood	Brazil	6978
GCA_003490605.1	6	ST-6 complex	10	14	4	5	4	7	8	NA	NA	NA	7404
GCA_003490645.1	724	ST-6 complex	10	20	21	5	4	7	8	NA	NA	NA	6920
GCA_003490755.1	6	ST-6 complex	10	14	4	5	4	7	8	NA	NA	NA	6939
GCA_003490865.1	54	ST-6 complex	10	14	22	5	4	7	22	NA	NA	NA	7243
GCA_003492045.1	95	ST-6 complex	31	14	4	5	4	7	8	NA	NA	NA	7676
GCA_003493435.1	55	ST-6 complex	10	14	4	5	4	31	8	NA	NA	NA	9981
GCA_003493725.1	637	ST-6 complex	10	14	5	7	4	12	8	NA	NA	NA	17920
GCA_003494005.1	53	ST-6 complex	10	14	5	7	4	7	8	NA	NA	NA	15694
GCA_003494495.1	119	ST-6 complex	10	14	4	5	4	47	8	NA	NA	NA	8606
GCA_003496985.1	117	ST-6 complex	10	14	4	3	4	7	8	NA	NA	NA	11874
GCA_003497005.1	1756	ST-222 complex	40	20	23	18	33	29	152	NA	NA	NA	25591
GCA_007896915.1	119	ST-6 complex	10	14	4	5	4	47	8	1983	blood	Norway	8465
GCA_016535385.1	44	ST-6 complex	10	14	4	3	4	3	8	NA	UCC Isolate	USA:MD	11163
GCA_019930705.1	44	ST-6 complex	10	14	4	3	4	3	8	NA	NA	Germany: Braunschweig	10761
GCA_021390955.1	6	ST-6 complex	10	14	4	5	4	7	8	1997/1998	blood	Brazil: Campinas, SP	6721
GCA_023223865.1	44	ST-6 complex	10	14	4	3	4	3	8	2007	throat swab	USA: Ann Arbor, MI	11223

Strain ID	MLST	CC	MLST alleles *adk*	*atpG*	*frdB*	*fucK*	*mdh*	*pgi*	*recA*	Collection Date	Source	Location	Core SNPs
GCA_023223885.1	44	ST-6 complex	10	14	4	3	4	3	8	2007	throat swab	USA: Ann Arbor, MI	11221
GCA_023223905.1	44	ST-6 complex	10	14	4	3	4	3	8	2007	throat swab	USA: Ann Arbor, MI	11217
GCA_023223925.1	44	ST-6 complex	10	14	4	3	4	3	8	2007	throat swab	USA: Ann Arbor, MI	11224
GCA_023224135.1	157	ST-6 complex	28	14	4	3	4	3	8	1984	eye swab	USA: Seattle, WA	11705
GCA_023224605.1	NA	None	28	14	38	3	4	3	8	1972/1981	ear fluid	USA: Nashville, TN	12103
GCA_023224875.1	6	ST-6 complex	10	14	4	5	4	7	8	1983	joint fluid	USA: Seattle, WA	3634
GCA_023224905.1	157	ST-6 complex	28	14	4	3	4	3	8	1978	joint fluid	USA: Boston, MA	12102
GCA_023224915.1	NA	None	28	14	4	3	4	3	43	1975	joint fluid	USA: Boston, MA	11693
GCA_023225125.1	6	ST-6 complex	10	14	4	5	4	7	8	1981	eye swab	USA: Seattle, WA	6984
GCA_023226125.1	44	ST-6 complex	10	14	4	3	4	3	8	1988	throat swab	USA: Seattle, WA	14095
GCA_023226525.1	157	ST-6 complex	28	14	4	3	4	3	8	1975	nasal swab	USA: Boston, MA	11813
GCA_026155805.1	6	ST-6 complex	10	14	4	5	4	7	8	2019	nasopharynx	Portugal	2448
GCA_900020195.1	190	ST-6 complex	10	14	4	5	85	7	8	2006/2012	CSF	Netherlands	3145
GCA_900022495.1	6	ST-6 complex	10	14	4	5	4	7	8	2006/2012	CSF	Netherlands	2522
GCA_900022515.1	6	ST-6 complex	10	14	4	5	4	7	8	2006/2012	blood	Netherlands	2519
GCA_900022525.1	6	ST-6 complex	10	14	4	5	4	7	8	2006/2012	blood	Netherlands	2683
GCA_900026985.1	6	ST-6 complex	10	14	4	5	4	7	8	2006/2012	CSF	Netherlands	2505
GCA_900026995.1	6	ST-6 complex	10	14	4	5	4	7	8	2006/2012	CSF	Netherlands	2519
GCA_900029875.1	6	ST-6 complex	10	14	4	5	4	7	8	2006/2012	blood	Netherlands	2505
GCA_900034945.1	6	ST-6 complex	10	14	4	5	4	7	8	2006/2012	blood	Netherlands	2522
GCA_900035265.1	6	ST-6 complex	10	14	4	5	4	7	8	2006/2012	blood	Netherlands	2743
GCA_900036645.1	6	ST-6 complex	10	14	4	5	4	7	8	2006/2012	CSF	Netherlands	2683
GCA_900041425.1	6	ST-6 complex	10	14	4	5	4	7	8	2006/2012	CSF	Netherlands	2743
GCA_900044695.1	190	ST-6 complex	10	14	4	5	85	7	8	2006/2012	blood	Netherlands	3145

Strain ID	MLST	CC	MLST alleles							Collection Date	Source	Location	Core SNPs
			adk	*atpG*	*frdB*	*fucK*	*mdh*	*pgi*	*recA*				
GCA_900407355.1	6	ST-6 complex	10	14	4	5	4	7	8	2010	CSF	Portugal	4582
GCA_900407455.1	6	ST-6 complex	10	14	4	5	4	7	8	2011	blood	Portugal	2476
GCA_900407525.1	190	ST-6 complex	10	14	4	5	85	7	8	2012	CSF	Portugal	4263
GCA_900407585.1	6	ST-6 complex	10	14	4	5	4	7	8	2013	blood	Portugal	2915
GCA_900407625.1	6	ST-6 complex	10	14	4	5	4	7	8	2014	blood	Portugal	2524
GCA_900407695.1	6	ST-6 complex	10	14	4	5	4	7	8	2016	blood	Portugal	4010
GCA_900407725.1	6	ST-6 complex	10	14	4	5	4	7	8	2015	blood	Portugal	2818
GCA_900407815.1	6	ST-6 complex	10	14	4	5	4	7	8	1992	CSF	Portugal	4210
GCA_900407835.1	6	ST-6 complex	10	14	4	5	4	7	8	1992	CSF	Portugal	3930
GCA_900407845.1	6	ST-6 complex	10	14	4	5	4	7	8	1993	CSF	Portugal	2589
GCA_900407865.1	282	ST-6 complex	10	14	5	44	4	7	8	1992	CSF	Portugal	15239
GCA_900407875.1	6	ST-6 complex	10	14	4	5	4	7	8	1992	CSF	Portugal	2412
GCA_900407885.1	6	ST-6 complex	10	14	4	5	4	7	8	1992	CSF	Portugal	2350
GCA_900407895.1	6	ST-6 complex	10	14	4	5	4	7	8	1993	CSF	Portugal	2593
GCA_900407965.1	6	ST-6 complex	10	14	4	5	4	7	8	1997	blood	Portugal	2526
GCA_900407985.1	6	ST-6 complex	10	14	4	5	4	7	8	1998	CSF	Portugal	3012
GCA_900407995.1	6	ST-6 complex	10	14	4	5	4	7	8	1997	CSF	Portugal	3168
GCA_900408005.1	6	ST-6 complex	10	14	4	5	4	7	8	1999	CSF	Portugal	2079
GCA_900408015.1	6	ST-6 complex	10	14	4	5	4	7	8	1998	blood	Portugal	3013
GCA_900408035.1	6	ST-6 complex	10	14	4	5	4	7	8	1998	blood	Portugal	2575
GCA_900408045.1	6	ST-6 complex	10	14	4	5	4	7	8	2000	blood	Portugal	2174
GCA_900408055.1	6	ST-6 complex	10	14	4	5	4	7	8	1999	blood	Portugal	2507
GCA_900408065.1	6	ST-6 complex	10	14	4	5	4	7	8	2001	blood	Portugal	2173
GCA_900408115.1	6	ST-6 complex	10	14	4	5	4	7	8	2003	blood	Portugal	3301

Strain ID	MLST	CC	MLST alleles *adk*	*atpG*	*frdB*	*fucK*	*mdh*	*pgi*	*recA*	Collection Date	Source	Location	Core SNPs
GCA_900408125.1	6	ST-6 complex	10	14	4	5	4	7	8	2004	blood	Portugal	2441
GCA_900408185.1	6	ST-6 complex	10	14	4	5	4	7	8	2007	blood	Portugal	2446
GCA_900478275.1	44	ST-6 complex	10	14	4	3	4	3	8	NA	NA	NA	10596
GCA_900635795.1	6	ST-6 complex	10	14	4	5	4	7	8	NA	NA	NA	2907
GCA_901472485.1	464	None	21	19	35	7	145	20	18	1940	Spinal fluid	NA	61606

*Collection dates are listed as years or year ranges based on available information. The final column presents SNP counts as determined by snippy-core, based on a comparison of all listed Hib genomes with strain AR2427 (CP148002) as the reference.

CC, clonal complex; ID, identification; MLST, multilocus sequence type; NA: not available; NCBI/GenBank, National Center for Biotechnology Information's GenBank database; SNP, single nucleotide polymorphism; ST, sequence type.

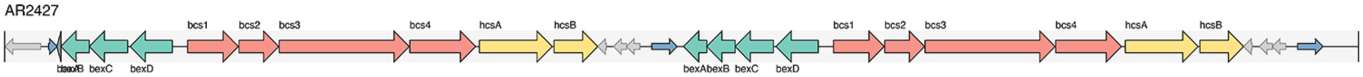

Appendix Figure. Schematic of the type b capsule synthesis locus for Hib strain AR2427 (genome CP148002.1; locus position nt 1830664–1863967) generated by hicap (https://github.com/scwatts/hicap). Note the presence of a truncated *bexA* gene at the far left but an intact *bexA-D* operon in green, close to the middle of the figure. Gene annotations are indicated. Blue arrows represent IS1016 sequences.
